# Comparative evaluation of imaging methods for prognosis assessment in esophageal squamous cell carcinoma: focus on diffusion-weighted magnetic resonance imaging, computed tomography and esophagography

**DOI:** 10.3389/fonc.2024.1397266

**Published:** 2024-07-03

**Authors:** Yang Li, Xiaohua Su, Yuguang Shang, Hui Liu, Weishuai Wang, Andu Zhang, Gaofeng Shi

**Affiliations:** ^1^ Department of Computed Tomography and Magnetic Resonance Imaging, The Fourth Hospital of Hebei Medical University, Shijiazhuang, China; ^2^ Department of Oncology, Hebei General Hospital, Shijiazhuang, China; ^3^ Department of Radiotherapy, The Fourth Hospital of Hebei Medical University, Shijiazhuang, China; ^4^ CS Service AP, Siemens Healthineers Digital Health Technology (Shanghai) Co., Ltd. Beijing Branch, Beijing, China

**Keywords:** esophageal cancer, radiotherapy, diffusion-weighted imaging, esophagography, computer tomography

## Abstract

**Objective:**

To identify the most sensitive imaging examination method to evaluate the prognosis of esophageal squamous cell carcinoma (ESCC).

**Materials and methods:**

Thirty patients with esophageal squamous cell carcinoma (ESCC) participated in the study and underwent chemoradiotherapy (CRT). They were divided into two groups based on their survival status: the survival group and non-survival group. The diagnostic tests were utilized to determine the most effective imaging examination method for assessing the prognosis.

**Results:**

1. There were no significant differences in tumor length shown on esophagography or computed tomography (CT) or the maximal esophageal wall thickness shown on CT at the specified time points between the two groups. 2. The tumor length on diffusion-weighted imaging (DWI) in the survival group was significantly lower than in the non-survival group at the end of the sixth week of treatment (P=0.001). The area under the ROC curve was 0.840 (P=0.002), and the diagnostic efficiency was moderately accurate. 3. The apparent diffusion coefficient (ADC) values of the survival group were significantly higher than those in the non-survival group at the end of the fourth week and sixth week of treatment (both P<0.001). Areas under the curve were 0.866 and 0.970, with P values of 0.001 and <0.001 and good diagnostic accuracy. Cox regression analyses indicated the ADC at the end of the sixth week of treatment was an independent risk factor.

**Conclusions:**

Compared with esophagography and CT, DW-MRI has certain advantages in predicting the prognosis of ESCC.

## 1 Introduction

Esophageal cancer (EC) represents a severe malignancy mainly due to its poor prognosis and survival rate, ranking sixth among all cancers in terms of mortality and eighth among the most commonly occurring cancers on a global scale (1).Since EC patients can only be diagnosed when they present with symptoms such as dysphagia, dysphagia, anemia, or weight loss, chemoradiotherapy (CRT) is widely used in cases of unresectable EC (2, 3). Thus, it has been deemed necessary to conduct a close assessment of the efficacy of CRT, which is very important for the adjustment of individualized treatment strategies for patients. Various methods are used to assess tumor response, and esophagography and computed tomography (CT) are routine tools for the evaluation of esophageal tumor response to treatment (4, 5). The criteria that are currently widely used in China to evaluate the efficacy of radiotherapy for EC follow the evaluation criteria for the efficacy of EC proposed by Professor Wan Jun in 1989, which are based on esophagography.

Esophagography is a noninvasive and inexpensive examination that details the structure and function of the esophageal mucosa and can determine the scope of esophageal lesions. Although it has wide availability, low cost, and rapid performance, there are also apparent limitations of this examination: esophagography cannot be used to evaluate the thickness of the esophageal wall or regional lymph node metastasis (6). However, the internationally standard evaluation criteria for the therapeutic effect of solid tumors, the Response Evaluation Criteria in Solid Tumors (RECIST) standard version 1.1 (7), are not entirely suitable for evaluating the efficacy of esophageal tumors after CRT.

CT can clearly show the thickness of the esophageal wall, tumor invasion and lymph node, and distant metastasis. However, because esophageal tumors originate in cavity organs, the esophageal structure still exists after CRT. There is edema in the wall of the esophagus after radiotherapy; CT is unable to differentiate between viable tumors, inflammatory changes, and scar tissue (8). CT is not sensitive enough to accurately evaluate treatment response. Functional imaging technology can compensate for the deficiency of morphological imaging technology and reflect the functional metabolism of tumor cells before morphological changes; it also has advantages for evaluating the efficacy of malignant tumors (9, 10).

Diffusion-weighted magnetic resonance imaging (DW-MRI) is an evolving imaging technique that contributes considerably and positively to the treatment of EC (11, 12). The apparent diffusion coefficient (ADC) is calculated for each pixel in an image, exhibited by a parametric map (13). The ADC is a reliable and reproducible value that serves as a promising noninvasive indicator that can be used to assess tumor aggressiveness as well as tumor responses to CRT (14, 15). The potential of the ADC value as a helpful marker has been well documented in various studies, which have highlighted its ability to predict treatment response and the survival probabilities of patients with esophageal squamous cell carcinoma (ESCC) (16).

This study analyzed the length of lesions shown on esophagography, CT, and DWI, the maximal esophageal wall thickness shown on CT, and the ADC values measured in DWI at specified time points, combined with diagnostic tests, between the survival group and the non-survival group to determine the most sensitive imaging examination method to evaluate the prognosis of patients with ESCC and provide valuable reference information for clinical work.

## 2 Material and methods

### 2.1 Patient selection criteria

All enrolled patients were diagnosed with ESCC by pathology and had an Eastern Cooperative Oncology Group of 0~2 (ECOG, which used to evaluate the patient’s performance status). All patients had no previous history of cancer or diseases that may affect the completion of treatment. No age limits were set. No distant metastases were found during routine imaging studies (MRI for the brain; CT for the lung, liver, and bone). All patients were first-time radiotherapy recipients. There were no MRI test contraindications, and patients approved all examinations.

### 2.2 Study population

A total of 30 patients with ESCC who were admitted to our hospital between February 2017 and June 2017 met the inclusion criteria. All patients were classified according to the 7th edition of the TNM staging system [International Union for Cancer Control (UICC)]. As of the follow-up date, all patients were divided into a survival group and a non-survival group according to their survival status. Details of the patients in the two groups are shown in **Table 1**.

**Table 1:** Characteristic of patients in survival group and non-survival group.

Characteristics	Survival group (n=12)	Non-Survival group (n=18)	Z/t	*P*
Gender, n (%)			4.678	<0.001^#^
Male	6 (50.0)	17 (94.4)		
Female	6 (50.0)	1 (5.6)		
Age	53-79 (66)	53-87 (70)	-0.811*	0.424^*^
Location, n (%)			3.202	0.002^#^
Cervical	1 (8.3)	0 (0)		
Upper	1 (8.3)	3 (16.7)		
Middle	9 (75.0)	14 (77.8)		
Lower	1 (8.3)	1 (5.6)		
T stage, n (%)				
T1-2	5 (41.7)	5 (27.8)	3.336	0.002^#^
T3	2 (16.7)	1 (5.6)		
T4	5 (41.7)	12 (66.7)		
N stage, n (%)			4.842	<0.001^#^
N0	2 (16.7)	0 (0.0)		
N1	5 (41.7)	6 (33.3)		
N2	5 (41.7)	12 (66.7)		
TNM stage, n (%)			3.981	<0.001^#^
I	2 (16.7)	0 (0.0)		
II	4 (33.3)	2 (11.1)		
III	6 (50.0)	16 (88.9)		
GTV volume (cm^3^), range (median)	18.65-272.31 (74.17)	25.14-284.20 (117.71)	-0.721*	0.477^*^
Dose (Gy), range (median)	50.4-60 (60)	50.4-60 (60)	0.335	0.787^#^

^#^, Mann-Whitney U test; ^*^, Student’s t-test.

### 2.3 Delineation of the target volume and organs at risk

Based on the CT images, the gross tumor volume of the primary tumor (GTV-p) and gross tumor volume of the metastatic lymph nodes (GTV-n) were outlined according to the department protocol on ESCC tissues. The OARs were also outlined in a manner consistent with international guidelines. Specifically, and in principle, CT images revealed that the standard GTV-p was a tumor size of more than 5 mm wide or an esophageal diameter of more than 10 mm with esophageal wall stiffness or full-wall thickening; the clinical target volume of the primary tumor (CTV-p) was contoured by extending 0.5 cm around the GTV-p in the axial direction and 2.0 cm in both the superior and inferior directions. Finally, the planning target volume of the primary tumor (PTV-p) was outlined around the CTV-p with a positive margin of 0.5 cm in the axial direction and 1.0 cm in both the superior and inferior directions. Afterwards, the GTV-n was defined as a paraesophageal lymph node with a short-axis diameter greater than 1.0 cm, and for lymph nodes in particular regions, such as those in the paraesophageal region or tracheoesophageal groove and cardiophrenic angle lymph nodes, the standard guideline was a short-axis diameter greater than 0.5 cm (17, 18). The PTV-n is the result of uniformly extending the GTV-n by 1.0 cm.

### 2.4 Treatment plan and delivery

The prescription radiation doses for the CRT group ranged from 50.4 to 60 Gy (median 60 Gy). All patients received intensity-modulated radiotherapy (IMRT), and all treatment plans included 1.8–2.0 Gy per fraction, 5 fractions per week, with a treatment time of 6 weeks. Each treatment plan required that the dose received by 95% of the PTV (PTV D95) be more than 100% of the prescription dose. The OAR doses were limited to lung V_5_ ≤ 65%, V_20_ ≤ 30%, and V_30_ ≤ 20%, heart_mean_ ≤ 30 Gy and cord_max_<45 Gy. The treatment plan was completed by a physiotherapist, as required, and confirmed by a superior physician. All patients completed the treatment plan.

### 2.5 Chemotherapy

The standard chemotherapy regimens were as follows: FP: cisplatin 25 mg/m2 × 3 days, 5-FU 450–500 mg/m2 × 5 days; and TP: cisplatin 25 mg/m2 × 3 days, paclitaxel 135-150 mg/m2 × 1 day.

### 2.6 Observation indicators

The length of lesions shown on esophagography, chest CT and DWI, the maximal esophageal wall thickness shown on chest CT and the ADC values shown on DWI of the two groups were measured before CRT, at the end of the second week of treatment, at the end of the fourth week of treatment and the end of the sixth week of treatment.

#### 2.6.1 Tumor length on esophagography

All patients were examined with esophagography at the specified time points. The tumor length on esophagography was measured on the axis image.

#### 2.6.2 Tumor length and maximal esophageal wall thickness on chest CT

All patients underwent CT at the indicated time points. Tumor length on CT was measured on a section showing the tumor in its entirety in the sagittal position. The maximum esophageal wall thickness of the tumor in the same horizontal region on the transverse section was measured based on the location of the tumor prior to CRT and the corresponding anatomic landmarks.

#### 2.6.3 Tumor length on DWI and ADC measurements

All patients underwent DW-MRI at the specified time points. The MRI examination involved a Siemens 3.0 T MRI scanner (Siemens Healthineers), an 18-channel body coil and a scanning sequence, including T1-weighted imaging (T1WI), T2-weighted imaging (T2WI) and DWI sequences. The b values (dispersion−sensitive gradient) were as follows (19–21): 0 and 600 s/mm^2^. The length of the lesions was measured on the axis image based on the DWI display signal. The images segmentation and measurement were performed using ITK-SNAP software version 3.8.0 (http://www.itksnap.org). Before delineating the tumor boundaries, we carefully reviewed the images across different sequences and selected the images of b-value with max image contrast between the lesion and background tissue. To address the issue of image clarity, we adjusted the window width and window level to show the lesion better (**Table 2**). Then, the lesion with high signal intensity was delineated layer by layer on the selected relatively high b-value. The ADC measurement process as follows: Using DWI images with b-values of 0 and 600 s/mm², we reconstructed the ADC maps. Both the reconstructed ADC maps and the original b=600 images were imported into the ITK-SNAP software. In ITK-SNAP, we meticulously delineated the tumor boundaries on the relatively high b-value images (b=600) layer by layer. These delineated regions of interest (ROIs) were then overlaid onto the ADC maps to measure the ADC values accurately.

**Table 2:** MRI scan sequences and parameters.

Parameters	Sequences		
	T1WI (Axial)	T2WI (Axial)	DWI (Axial)
TR (ms)	4.00	3000	8200
TE (ms)	1.29	81	49
FOV Read (mm)	380	380	300
FOV Phase (%)	81.3	100	68
Slice Thickness (mm)	3.5	6	3
Distance Factor (%)	20	20	25
Base Resolution	320	320	100
Phase Resolution (%)	75	/	100
Phase Oversampling (%)	30	37.5	40
Slicer Oversampling (%)	33.3	/	/
Average	1	1	2
b-value (s/mm^2^)	/	/	600
Phase encoding	A>>P	R>>L	A>>P
Blade coverage (%)	/	100	/
Bandwidth (Hz)	1040	781	2272
RF Pulse Mode	Fast	Fast	Normal
Turbo Factor	/	43	/
Echo spacing (ms)	/	3.66	0.54
Fat-Water Contrast	Dixon	SPAIR	Fat Saturation

All of the above imaging data were analyzed by two experienced radiologists (with 10 and 8 years of experience in clinical radiology, respectively) who reached a consensus. In order to assess the intra-observer reproducibility of the measurements, a concordance analysis of 50 images (10 images per observation indicator) was performed using the values measured by two radiologists. The ICC interpretations were as follows: excellent (ICC ≥ 0.90); good (0.75 ≤ ICC < 0.90); moderate (0.50 ≤ ICC < 0.75); and poor (ICC < 0.50), and the ICCs are 0.98 (tumor length on esophagography), 0.83 (tumor length on CT), 0.98 (maximal esophageal wall thickness on CT), 0.94 (tumor length on DWI) and 0.90 (ADC), respectively, with the p values < 0.05, and the above results indicate good consistency and repeatability. Final analysis was performed using the average of the values measured by the two radiologists.

### 2.7 Statistical methods

Statistical analysis was performed using GraphPad software (GraphPad Prism v7.0, GraphPad Software). The normality of quantitative data was assessed using a Kolmogorov-Smirnov test. Categorical variables and continuous data conforming to a nonnormal distribution were analyzed using the nonparametric Mann-Whitney U test, while continuous data conforming to a normal distribution were analyzed using Student’s t-test. The variables at different time points were determined using the repeated measures analysis of variance (ANOVA). Cox regression models and Kaplan-Meier analyses were conducted to estimate overall survival (OS), and the log-rank test was applied to assess differences between groups. P<0.05 was considered statistically significant. Receiver operating characteristic (ROC) curves were plotted for the observation indicators, and the area under the curve (AUC) was calculated. The optimal cutoff values were chosen as follows: when Youden’s index (YI=sensitivity+specificity-1) was the maximum, AUC values between 0.9 and 1.0 were deemed “accurate”; 0.7-0.9 “moderately accurate”; and 0.5-0.7 “uninformative” (22, 23).

## 3 Results

### 3.1 OS and local control

As of January 1, 2021, all patients were followed up for over 3 years, no patients were lost to follow-up, and the total follow-up rate was 100%. There were 12 patients in the survival group and 18 in the non-survival group. OS was evaluated from the date of radiotherapy to the date of death or the last follow-up. All patients’ 1- and 3-year OS rates were 80.0% and 43.3%, respectively, with a median survival time of 30 months. All patients’ 1- and 3-year LC rates were 83.2% and 50.6%, respectively, with a median LC time of 31 months.

### 3.2 Tumor length shown on esophagography

**Figure 1:**
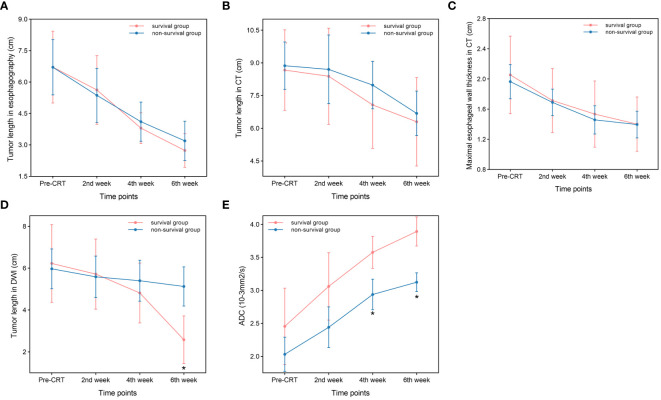
Comparison between the survival group and non-survival group. **(A)** Tumor length in esophagography. **(B)** Tumor length in CT. **(C)** Maximal esophageal wall thickness in CT. **(D)** Tumor length in DWI (P=0.001,at the end of the sixth week). **(E)** ADC. (P<0.001,at the end of the fourth and sixth week) *p<0.05.

Tumor lengths shown on esophagography in all patients before CRT, at the end of the second week of treatment, at the end of the fourth week of treatment, and at the end of the sixth week of treatment were 6.71 ± 2.63 cm, 5.47 ± 2.55 cm, 3.98 ± 1.62 cm, and 3.01 ± 1.65 cm, respectively. Tumor length varied at different points in the survival group (F=16.897, P<0.001) and in the non-survival group (F=37.782, P<0.001). With increased radiotherapy sessions, tumor length showed a sustained and rapid downward trend in both groups, and no significant difference in tumor length was shown on esophagography between the two groups at the specified time points (**Figure 1A**). Diagnostic tests were carried out at the specified time points to assess tumor length on esophagography. The ROC curve showed that the P value was greater than 0.05, and the area under the curve was between 0.484-0.569 (**Table 3**), indicating that the diagnostic effectiveness of tumor length measured on esophagography was low.

**Table 3:** ROC curve analysis.

Observation indicators	AUC	P	95% CI	
			lower	Upper
Tumor length in esophagography (cm)				
before CRT	0.484	0.882	0.258	0.710
2nd week	0.484	0.882	0.263	0.704
4th week	0.569	0.525	0.357	0.782
6th week	0.523	0.832	0.310	0.736
Tumor length in CT (cm)				
before CRT	0.521	0.849	0.299	0.743
2nd week	0.495	0.966	0.270	0.720
4th week	0.602	0.352	0.383	0.821
6th week	0.539	0.719	0.311	0.768
The maximal esophageal wall thickness in CT (cm)				
before CRT	0.500	1.000	0.264	0.736
2nd week	0.495	0.966	0.263	0.727
4th week	0.498	0.983	0.268	0.727
6th week	0.465	0.751	0.244	0.686
Tumor length in DWI (cm)				
before CRT	0.479	0.849	0.249	0.709
2nd week	0.477	0.832	0.249	0.705
4th week	0.569	0.525	0.350	0.789
6th week	0.840	0.002	0.700	0.980
ADC (10^-3^mm^2^/s)				
before CRT	0.576	0.485	0.340	0.813
2nd week	0.745	0.025	0.560	0.931
4th week	0.866	0.001	0.740	0.992
6th week	0.970	<0.001	0.918	1.000

2nd week, the end of the second week.4th week, the end of the fourth week. 6th week, the end of the sixth week.

### 3.3 Tumor length shown on chest CT

Tumor lengths on chest CT in all patients before CRT, at the end of the second week of treatment, at the end of the fourth week of treatment, and the end of the sixth week of treatment were 8.79 ± 2.45 cm, 8.58 ± 3.24 cm, 7.62 ± 2.59 cm, and 6.53 ± 2.53 cm, respectively. Tumor length varied at different points in the survival group (F=6.820, P=0.001) and in the non-survival group (F=7.005, P=0.002). With an increase in radiotherapy sessions, tumor length on CT showed a sustained and rapid downward trend in both groups, and there was no significant difference in tumor length shown on CT between the two groups at the specified time points (**Figure 1B**). Diagnostic tests were carried out at the specified time points for tumor length on CT. The ROC curve showed that the P value was greater than 0.05, and the area under the curve was between 0.495 and 0.602 (**Table 3**), indicating that the diagnostic effectiveness of tumor length shown on chest CT was low.

### 3.4 Maximal esophageal wall thickness shown on chest CT

The maximal esophageal wall thicknesses shown on chest CT in all patients before CRT, at the end of the second week of treatment, at the end of the fourth week of treatment, and at the end of the sixth week of treatment were 2.00 ± 0.61 cm, 1.70 ± 0.49 cm, 1.49 ± 0.52 cm, and 1.40 ± 0.44 cm, respectively. The maximal esophageal wall thickness varied at different points in the survival group (F=17.775, P<0.001) and in the non-survival group (F=58.602, P<0.001). With an increase in the number of radiotherapy sessions, the maximal esophageal wall thickness shown on CT initially showed a sustained and rapid downward trend, which slowed in the fifth week and sixth week in both groups, and there was no significant difference in the maximal esophageal wall thickness shown on chest CT between the two groups at the specified time points (**Figure 1C**). The ROC curve showed that the P value was greater than 0.05, and the area under the curve was between 0.465-0.500 (**Table 3**), indicating that the diagnostic effectiveness of the maximal esophageal wall thickness shown on CT was low.

### 3.5 Tumor length shown on DW-MRI

**Figure 2:**
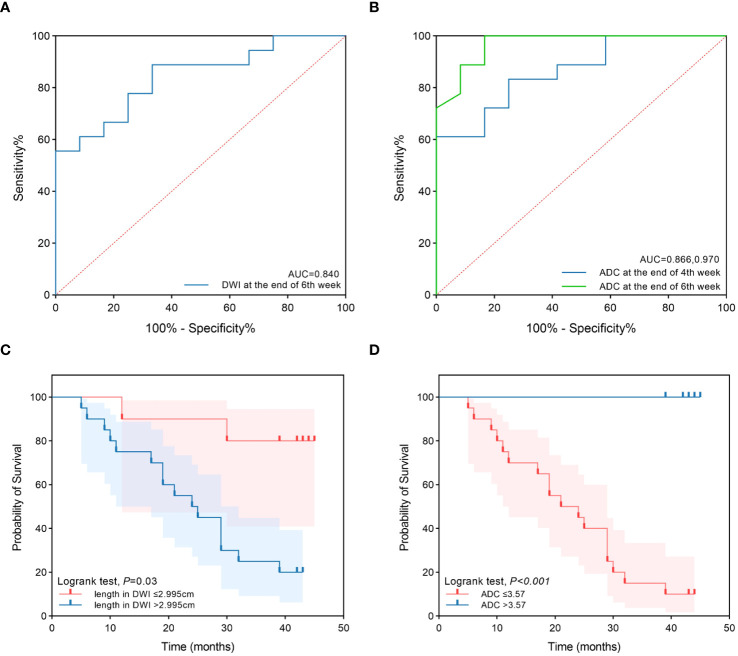
The ROC curves and survival curves. **(A)** The ROC curves of tumor length in DWI at the end of 6th week of treatment to predict prognosis. **(B)** The ROC curves of ADC at the end of 4th and 6th week of treatment to predict prognosis. **(C)** The survival curves of the two groups for tumor length in DWI at the end of 6th week ≤2.995cm and >2.995cm. **(D)** The survival curves of the two groups for ADC at the end of 6th week ≤3.57×10^-3^mm^2^/s and >3.57×10-3mm^2^/s.

Tumor lengths shown on DW-MRI in all patients before CRT, at the end of the second week of treatment, at the end of the fourth week of treatment, and at the end of the sixth week of treatment were 6.07 ± 2.32 cm, 5.64 ± 2.23 cm, 5.17 ± 2.07 cm, and 4.11 ± 2.22 cm, respectively. Three patients did not have a high signal expression on DWI at the end of the sixth week of treatment, all of whom had long-term survival, with survival times of 39 months, 42 months, and 43 months. Tumor length shown on DWI varied at different points in the survival group (F=21.379, P<0.001) but not in the non-survival group (F=3.146, P=0.057). With an increase in the number of radiotherapy sessions, tumor length shown on DWI showed a continuous and rapid declining trend, which was more evident after the fourth week in the survival group. In addition, three patients did not have a high signal expression on DWI at the end of treatment. Tumor length on DWI increased slightly in the second week compared to pretreatment and then showed a slow downward trend in the non-survival group. All patients in this group had a high signal expression on DWI at the end of treatment (**Figure 1D**). There was no significant difference in tumor length shown on DWI between the two groups before CRT, at the end of the second week of treatment, at the end of the fourth week of treatment, and tumor length in the survival group was significantly lower than that in the non-survival group at the end of the sixth week of treatment (t=-3.687, P=0.001). Diagnostic tests were carried out at the specified time points for tumor length on DWI. The ROC curve showed that with tumor length shown on DWI as the diagnostic index at the end of the sixth week of treatment, the P value was 0.002, and the area under the curve was 0.840. The diagnostic efficacy was moderately accurate, with a cutoff value of 2.995 cm, a sensitivity of 0.889, and a specificity of 0.667 (**Figure 2A**, **Table 3**). With tumor length shown on DWI at the end of the sixth week of treatment as the cutoff point to divide the whole group of patients into two groups (2.995 cm), there were ten patients with a tumor length ≤2.995 cm and 20 patients with a tumor length > 2.995 cm. The 1- and 3-year OS rates of the two groups were 90.0% and 80.0% and 75.0% and 25.0%, respectively, with medium survival times of 40 months and 24 months, respectively (χ^2 =^ 8.531, P=0.003) (**Figure 2C**).

### 3.6 ADC on DWI

The ADC values on DWI in all patients before CRT, at the end of the second week of treatment, at the end of the fourth week of treatment and the end of the sixth week of treatment were 2.20 ± 0.72×10-3 mm^2^/s, 2.69 ± 0.75×10-3 mm^2^/s, 3.19 ± 0.53×10-3 mm^2^/s, and 3.43 ± 0.49×10-3 mm^2^/s, respectively. The ADC values differed at different time points in the survival group (F=18.939, P<0.001) and the non-survival group (F=45.122, P<0.001). With an increase in radiotherapy sessions, the ADC value showed a continuous upward trend in the survival and non-survival groups. However, the upward trend in the non-survival group slowed significantly from the fourth week. There were no significant differences in ADC values between the two groups before CRT and at the end of the second week of treatment. The ADC value of the survival group was significantly higher than that of the non-survival group at the end of the fourth week and sixth week of treatment (t=3.942, 6.592, P<0.001, P<0.001) (**Figure 1E**). Diagnostic tests were carried out at the specified time points for the ADC value. The ROC curve showed that with the ADC at the end of the fourth week of treatment and the end of the sixth week of treatment as the diagnostic index, the P values were 0.001 and <0.001, the areas under the curve were 0.866 and 0.970, and the diagnostic efficacies were moderately accurate and accurate, with cutoff values of 2.965×10-3 mm2/s and 3.570×10^-3^ mm^2^/s, sensitivities of 1 and 0.833, and specificities of 0.611 and 1 (**Figure 2B**, **Table 3**). With the ADC at the end of the sixth week of treatment as the cutoff point to divide the whole group of patients into two groups (3.570×10-3 mm^2^/s), there were 20 patients with a tumor length ≤ 3.570×10-3 mm2/s and ten patients with a tumor length >3.570×10-3 mm^2^/s. The 1- and 3-year OS rates of the two groups were 70.0% and 15.0% and 100.0% and 100.0%, respectively, with medium survival times of 23 months and 42 months, respectively (χ^2 =^ 18.843, P<0.001) (**Figure 2D**).

### 3.7 Cox regression analysis

Considering the potential clinical significance of the observation indicators for OS, we aimed to clarify the correlations of observation indicators with other traditional clinical features, including age, sex, tumor site, TNM stage, GTV, and prescription dose. The observation indicators were initially merged with other variables, and Cox analysis was subsequently performed. Then, univariate Cox analysis indicated that sex (*P*=0.035), TNM stage (*P*=0.051), tumor length on DWI at the end of the sixth week of treatment (*P*=0.001), and ADC at the end of the sixth week of treatment (*P* < 0.001) were all risk factors. Nonetheless, the ADC at the end of the sixth week of treatment (*P*=0.05) retained significance in the multivariate Cox regression analysis (**Table 4**).

**Table 4:** Univariate and multivariate Cox analysis for clinical characteristics.

Variables	Univariate Cox analysis				Multivariate Cox analysis			
	HR	95.0% CI		P	HR	95.0% CI		P
		Lower	Upper			Lower	Upper	
Sex	0.114	0.015	0.860	0.035	0.182	0.020	1.647	0.130
Age	1.034	0.967	1.105	0.327	–	–	–	–
TNM	3.857	0.996	14.931	0.051	2.171	0.454	10.392	0.332
Site	1.316	0.575	3.014	0.515	–	–	–	–
GTV volume	1.002	0.996	1.009	0.503	–	–	–	–
Dose	1.006	0.886	1.142	0.930	–	–	–	–
Tumor length in esophagography (6th)	1.010	0.785	1.299	0.940	–	–	–	–
Tumor length in CT(6th)	1.060	0.887	1.267	0.521	–	–	–	–
The maximal esophageal wall thickness in CT (6th)	1.050	0.389	2.833	0.923	–	–	–	–
Tumor length in DWI (6th)	1.489	1.168	1.900	0.001	0.937	0.637	1.378	0.742
ADC (6^th^)	0.069	0.019	0.252	<0.001	0.146	0.021	1.002	0.05

6th week, the end of the sixth week.

### 3.8 Diagrams of typical cases

**Figure 3:**
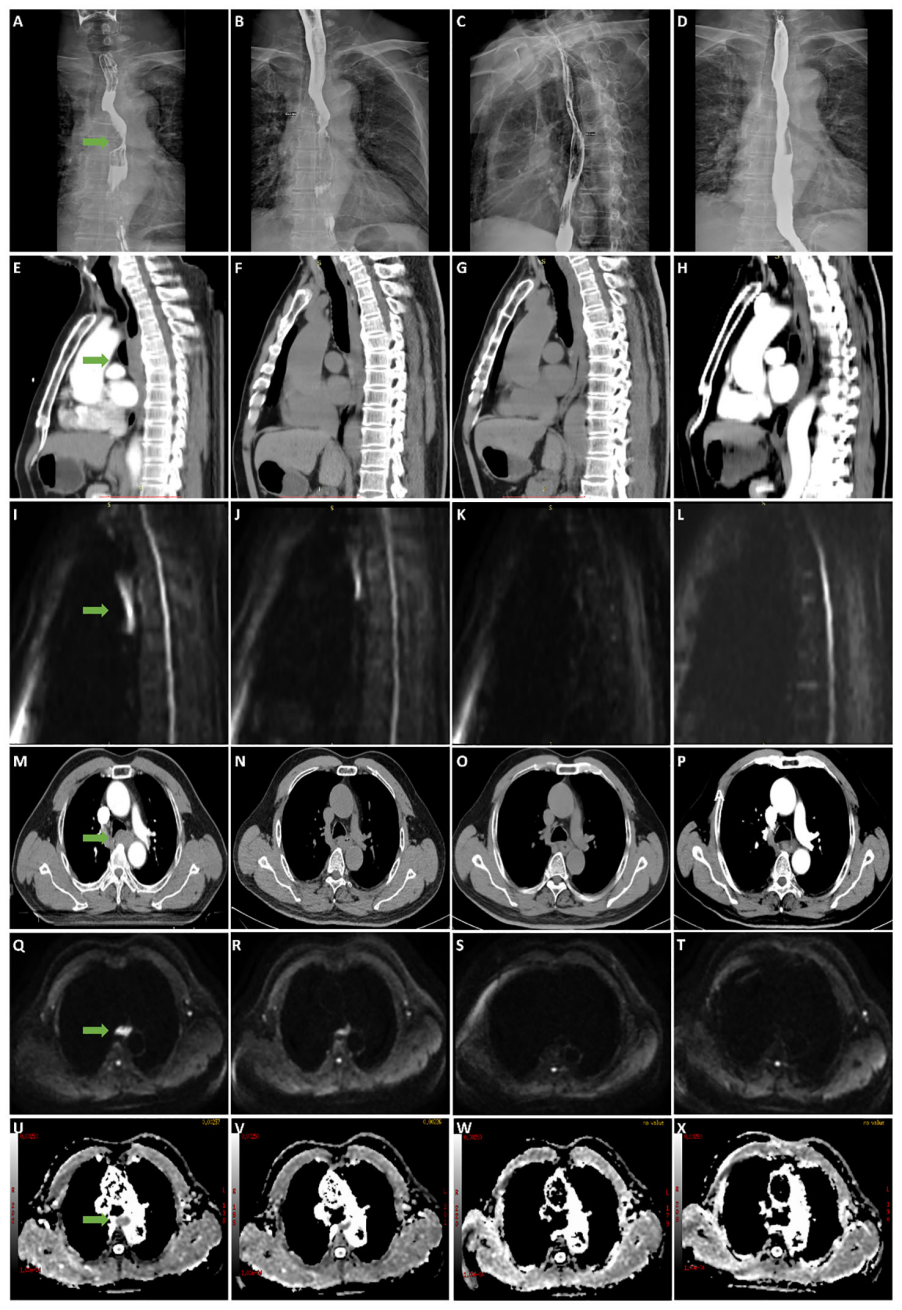
** **A 63-year-old male patient with stage III esophageal cancer in survival group was monitored for treatment response using esophagography, CT, and DWI at various stages: before CRT, and at the end of the 2nd, 4th, and 6th weeks of treatment. **(A–D)** Tumor in esophagography images. **(E–H)** Tumor in CT images (median sagittal section). **(I–L)** Tumor in DWI images (median sagittal section). **(M–P)** Tumor in CT images (transverse section). **(Q–T)** Tumor in DWI images (transverse section). **(U–X)** Tumor in ADC images (transverse section).

**Figure 4:**
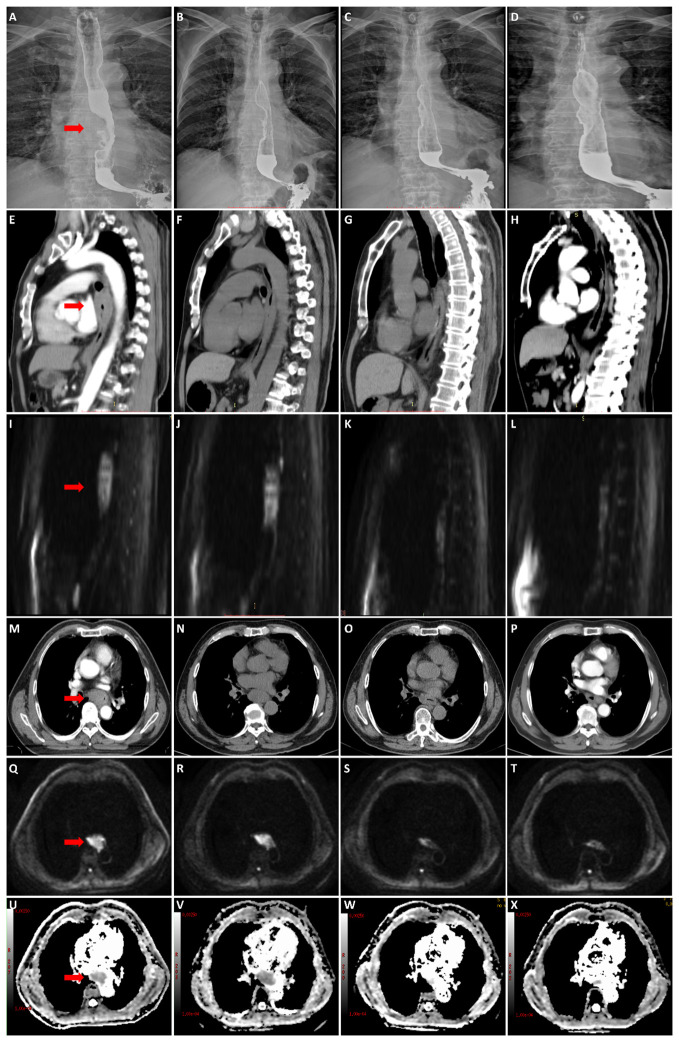
A 57-year-old male patient with stage III esophageal cancer in non-survival group was monitored for treatment response using esophagography, CT, and DWI at various stages: before CRT, and at the end of the 2nd, 4th, and 6th weeks of treatment. **(A–D)** Tumor in esophagography images. **(E–H)** Tumor in CT images (median sagittal section). **(I–L)** Tumor in DWI images (median sagittal section). **(M–P)** Tumor in CT images (transverse section). **(Q–T)** Tumor in DWI images (transverse section). **(U–X)** Tumor in ADC images (transverse section).

**Figure 3** show the esophagography, CT, and DW images of one patient in the survival group before CRT, at the end of the second week of treatment, at the end of the fourth week of treatment, and at the end of the sixth week. **Figure 4** shows the esophagography, CT, and DW images of one patient in the non-survival group at the specified time points.

## 4 Discussion

Malignant tumors are a severe primary disease that threatens human health and social development. EC is one of the leading causes of cancer-related death in China (24, 25); its incidence has prominent regional distribution characteristics, and ESCC is the primary tissue type (26, 27). It is of great significance to evaluate the therapeutic effect of malignant tumors objectively, quantitatively, and accurately, and prognostic indicators could guide individualized treatment decisions and thus improve the benefits of treatment. However, most methods, including CT, esophagography, endoscopic biopsy, and endoscopic ultrasonography (EUS), yield unsatisfactory results for tumor response to neoadjuvant CRT (nCRT) (28–30). Metabolic and functional imaging modalities such as 18F-fluorodeoxyglucose positron emission tomography integrated with CT (18F-FDG PET/CT) and DW-MRI may be more promising because they allow the biological and microstructural characterization of tumors and visualization of treatment-induced changes before volumetric changes become apparent (31, 32). However, PET/CT is expensive and not widely used, while the use of MRI in the treatment response evaluation of ECs has gained increasing interest. DWI can afford valuable markers to predict treatment response, as well as the survival of patients with ESCC, and the sustained high signal expression on DWI is a risk factor (33). However, high signal expression judgment is relatively subjective. Physicians with different levels of experience and qualifications may define high, slightly higher, and no signals differently. Hence, this study aimed to find a more objective, more straightforward, and more sensitive imaging examination method to judge the prognosis of ESCC and provide valuable reference information for clinical use.

Here, both the survival and the non-survival groups had similar trends in tumor length measured on esophagography before CRT and at the end of the second week, fourth week, and sixth week of treatment: a monotonically decreasing trend. However, there was no significant difference in tumor length measured on esophagography between the two groups at the four-time points. The ROC curve showed that the P-values were greater than 0.05, indicating that the prognostic diagnostic efficiency of tumor length is very low compared to that measured on esophagography. Similar to the trend of tumor length measured on esophagography, the trends of tumor length measured on CT and the maximal esophageal wall thickness measured on CT in the survival group were similar to those in the non-survival group, and the ROC curves showed that the P-values were greater than 0.05, indicating that according to tumor length measured on CT and the maximal esophageal wall thickness measured on CT, their prognostic diagnostic efficiencies were very low. Therefore, traditional morphological imaging techniques are unreliable in the early evaluation of tumor response to CRT (34, 35), making it unreasonable to determine the downgrade or upgrade of treatment.

Tumor length measured on DWI at different points showed a sustained rapid declining trend, which was more pronounced after four weeks of treatment in the survival group, and a slow downward trend was observed in the non-survival group. Three patients did not have a high signal expression on DW images at the end of the sixth week of treatment, all of whom achieved long-term survival, and all the patients in the non-survival group had high signal expression at the end of radiotherapy. Tumor length measured on DWI in the survival group was significantly lower than that in the non-survival group at the end of the sixth week of treatment (P=0.001). The ROC curve showed that according to tumor length measured on DWI at the end of the sixth week of treatment as the diagnostic index (2.995 cm), the area under the curve was 0.840. The diagnostic efficiency was accurate, with a sensitivity of 0.889 and a specificity of 0.667. The whole group was divided into two groups according to tumor length measured on DWI at the end of the sixth week of treatment as the cutoff value (2.995 cm): 10 patients had a tumor length ≤2.995 cm, 20 patients had a tumor length >2.995 cm, and the 1- and 3-year survival rates in both subgroups were 90.0% and 80.0% and 75.0% and 25.0% (P=0.003), which indicated that tumor length measured on DWI could effectively predict prognosis. This is similar to the conclusions of other studies (19).

The ADC is inversely correlated with tissue cellularity. Cytotoxic therapy affects the permeability and integrity of the tumor cell membrane. It induces apoptosis, necrosis, and dissolution, leading to changes in tissue density and water molecule dispersion, causing increased ADC values. The ADC has emerged as a potential biomarker of response to cancer therapy (36, 37). Many studies (38–40) have confirmed that the ADC value increases significantly after CRT for EC: compared with those who did not respond well, there was a significant increase in the ADC values after antitumor therapy in those who did respond well. This study showed that the ADC value of the whole group of patients also showed a gradual upward trend after treatment. However, with an increase in the frequency of radiotherapy, the ADC value showed a continuous upward trend in the survival group. In contrast, the rising trend decreased from the end of the fourth week of treatment in the non-survival group. There were significant differences between the two groups at the end of the fourth week and sixth week of treatment, and the ADC value of the survival group was significantly higher than that of the non-survival group (P<0.001). The ROC curve showed that with the ADCs at the end of the fourth week of treatment and the end of the sixth week of treatment as the diagnostic indexes (2.965×10-3 mm^2^/s and 3.570×10-3 mm^2^/s), the P values were 0.001 and <0.001, and the areas under the curve were 0.866 and 0.970, with sensitivities of 1 and 0.833 and specificities of 0.611 and 1. The diagnostic efficacies were accurate; therefore, the diagnostic indicator of the ADC at the end of the sixth week of treatment was better. With the ADC at the end of the sixth week of treatment as the cutoff point to divide the whole group of patients into two groups (3.570×10-3 mm^2^/s), the OS rate of the ADC>3.570×10-3 mm^2^/s group was significantly better than that of the ADC≤ 3.570×10^-3^ mm^2^/s group (P<0.001). Univariate and multivariate Cox regression analyses also indicated that the ADC at the end of the sixth week of treatment was a risk factor, similar to other reports (33). From the diagrams of the two patients, we can see the uniqueness and advantages of functional imaging technology, the maximum wall thickness of the patients in the survival group gradually decreased throughout the treatment. By the end of the treatment, no high signal was observed on the DW images, and the lesions were no longer visible. In contrast, patients in the non-survival group showed a reduction in lesions on esophageal esophagography by the end of the treatment, with a significant decrease in maximum esophageal wall thickness and a shorter tumor length compared to before CRT. However, despite these reductions, the lesions still exhibited high signals on DW images at the end of the treatment. similarly, Alicia S Borggrevet (11) reported that early changes on 18F-FDG PET/CT and DW-MRI during nCRT could help identify EC patients who could achieve pathologic complete response. However, these changes cannot be observed with morphological imaging technology, the integration of DW-MRI into clinical practice for the management of esophageal cancer can provide significant advantages over traditional imaging methods. It offers superior prognostic value, enabling more precise and early predictions of survival outcomes, thus facilitating optimized therapeutic strategies. Moreover, the non-invasive nature of DWI makes it a favorable alternative to more invasive diagnostic procedures, thereby reducing patient discomfort and associated risks.

The results of this study showed that when tumor length measured on DWI at the end of the sixth week of treatment and the ADC values measured at the end of the fourth week and sixth week of treatment were used as diagnostic indicators, the prognosis can be effectively judged. Their diagnostic efficacies are better than those of morphological examination methods. Moreover, DWI is a simple, reliable, convenient, and sensitive prediction method that can be used to judge prognosis effectively. The strength of this study is that it allows for an intuitive comparison of the advantages and disadvantages of the three imaging methods by using dense time points. However, this study has some limitations. The first is the number of cases in our study was relatively small, which may affect the universality of the results, and differences in scanning equipment and parameter settings may lead to data variability. Therefore, larger scale, multicenter studies are needed in the future to validate our findings and further clarify the role of DWI/ADC values in the prognosis evaluation of esophageal cancer radiotherapy. Additionally, due to the limitations of scanning conditions at that time, we did not collect data that could be used for advanced diffusion models, including IVIM and DKI, have to some extent corrected potential flaws in DWI, such as susceptibility to cellular or vascular system effects (41), or the influence of nonnormally distributed motion on ADC values (42). Many scholars have studied advanced diffusion models such as IVIM and have drawn meaningful conclusions (43–45). In the future, we will attempt to use advanced diffusion models such as IVIM and DKI to conduct more in-depth research in this field.

In summary, functional imaging technology can accurately reflect the actual treatment effect of tumors. As a relatively economical and straightforward examination method, DW-MRI can allow the ADC to be measured directly on maps, reflecting the metabolic information of tissue cells, and can provide tumor-related information objectively and quantitatively, which is worthy of clinical application. Radiotherapists must be aware of the strengths and limitations of different imaging modalities in various clinical settings. If necessary, information from anatomic and functional imaging can be combined. A multimodality-based approach to imaging is essential in clinical practice to achieve the best possible outcome for patients with EC.

## 5 Conclusion

Compared with esophagography and CT, DW-MRI has certain advantages in predicting the prognosis of ESCC. ADC value as a non-invasive imaging biomarker, have the potential to predict the prognosis of esophageal cancer radiotherapy and provide valuable information for personalized treatment.

## Data availability statement

The original contributions presented in the study are included in the article/supplementary material. Further inquiries can be directed to the corresponding author. Requests to access these datasets should be directed to zhangandu@hebmu.edu.cn.

## Ethics statement

The studies involving humans were approved by The Fourth Hospital of Hebei Medical. The studies were conducted in accordance with the local legislation and institutional requirements. The participants provided their written informed consent to participate in this study.

## Author contributions

YL: Conceptualization, Data curation, Investigation, Methodology, Validation, Visualization, Writing – original draft, Writing – review & editing. XS: Conceptualization, Data curation, Investigation, Methodology, Validation, Visualization, Writing – original draft, Writing – review & editing. YS: Data curation, Investigation, Methodology, Resources, Validation, Writing – original draft. HL: Conceptualization, Data curation, Formal analysis, Methodology, Resources, Software, Validation, Visualization, Writing – original draft, Writing – review & editing. WW: Software, Methodology, Validation, Writing – original draft. AZ: Conceptualization, Data curation, Investigation, Methodology, Validation, Visualization, Writing – original draft, Writing – review & editing, Formal Analysis, Funding acquisition. GS: Conceptualization, Project administration, Resources, Writing – review & editing.
